# Novel HPLC Analysis of Hydrocortisone in Conventional and Controlled-Release Pharmaceutical Preparations

**DOI:** 10.1155/2017/9495732

**Published:** 2017-06-04

**Authors:** Ofosua Adi-Dako, Samuel Oppong Bekoe, Kwabena Ofori-Kwakye, Enoch Appiah, Paul Peprah

**Affiliations:** ^1^Department of Pharmaceutics, Faculty of Pharmacy and Pharmaceutical Sciences, Kwame Nkrumah University of Science and Technology (KNUST), Kumasi, Ghana; ^2^Department of Pharmaceutics and Microbiology, School of Pharmacy, University of Ghana, Legon, Accra, Ghana; ^3^Department of Pharmaceutical Chemistry, Faculty of Pharmacy and Pharmaceutical Sciences, Kwame Nkrumah University of Science and Technology (KNUST), Kumasi, Ghana

## Abstract

An isocratic sensitive and precise reverse phase high-performance liquid chromatography (RP-HPLC) method was developed and validated for the determination and quantification of hydrocortisone in controlled-release and conventional (tablets and injections) pharmaceutical preparations. Chromatographic separation was achieved on an ODS (C18), 5 *μ*m, 4.6 × 150 mm, with an isocratic elution using a freshly prepared mobile phase of composition methanol : water : acetic acid (60 : 30 : 10, v/v/v) at a flow rate of 1.0 ml/min. The detection of the drug was successfully achieved at a wavelength of 254 nm. The retention time obtained for the drug was 2.26 min. The proposed method produced linear detectable responses in the concentration range of 0.02 to 0.4 mg/ml of hydrocortisone. High recoveries of 98–101% were attained at concentration levels of 80%, 100%, and 120%. The intraday and interday precision (RSD) were 0.19–0.55% and 0.33–0.71%, respectively. A comparison of hydrocortisone analyses data from the developed method and the official USP method showed no significant difference (*p* > 0.05) at a 95% confidence interval. The method was successfully applied to the determination and quantification of hydrocortisone in six controlled-release and fifteen conventional release pharmaceutical preparations.

## 1. Introduction

Hydrocortisone is a naturally occurring corticosteroid hormone secreted by the adrenal cortex and released during times of stress. The synthetic drug is employed in the management of inflammatory and rheumatoid diseases, allergic conditions, and autoimmune disorders such as Addison's disease (adrenal insufficiency disease). Hydrocortisone is commercially available in pharmaceutical formulations such as tablets, capsules, creams, ointments, and injections. Hydrocortisone may exist commercially as the unchanged hormone or as the acetate, cypionate, sodium phosphate, butyrate, valerate, and the sodium succinate forms [1].

A search of the literature revealed that few analytical methods such as ultraviolet spectrophotometry [2], electrokinetic capillary chromatography [3, 4], photochemically enhanced fluorescence [5], thin-layer chromatography- (TLC-) densitometry [1, 6], and high-performance thin-layer chromatography (HPTLC) [7] have been used for the estimation of hydrocortisone in pharmaceutical formulations. However, modern chromatographic techniques such as high-performance liquid chromatography (HPLC) [8–12] and ultra-performance liquid chromatography (UPLC) [13] are preferentially employed for the determination of hydrocortisone in conventional pharmaceutical products such as tablets, creams, and injections. The different HPLC methods employ different chromatographic conditions including the use of complex and sometimes expensive solvent systems. Thus, access to some of these solvent systems in resource-poor countries in the developing countries can be challenging. The development of a simple and economical HPLC method using readily available reagents for the identification and quantification of hydrocortisone in pharmaceutical preparations would be an advantage in resource-poor countries.

The upsurge of autoimmune diseases such as adrenal insufficiency is a major health concern and the long-term use of conventional hydrocortisone tablets in the management of such a condition is problematic. The twice or thrice daily dosing of conventional hydrocortisone tablets to patients with adrenal insufficiency disease is incapable of mimicking the unique diurnal cortisol circadian pattern. Thus, most persons with adrenal insufficiency continue to suffer from poor therapeutic management resulting in poor quality of life and increased mortality. There is therefore the need for the development of creative and innovative treatment models for hydrocortisone replacement therapy in such dire situations. The use of controlled-release hydrocortisone oral dosage forms holds great promise with the capability to replicate the unique physiological pattern of hydrocortisone. In addition, such formulations are better able to manage and control the levels of morning androgen levels. The accurate monitoring of the quality of these promising new drug therapies is an important prerequisite to obtain quality healthcare [14]. The improved therapy will enhance patient compliance and ensure the delivery of controlled amounts of hydrocortisone at the absorption site compared to the immediate release pattern of conventional tablets [15].

The aim of this study was to develop and validate a simple, sensitive, and reproducible isocratic reverse phase HPLC method with ultraviolet/visible (UV) detection capable of determination and quantification of hydrocortisone in conventional and controlled-release pharmaceutical preparations. The application of the method in determining the content of hydrocortisone in controlled-release and commercially available conventional dosage forms was also studied.

## 2. Materials and Methods

### 2.1. Standards and Reagents

The reference material of hydrocortisone (Sigma Aldrich, USA), HPLC grade methanol (Fisher Scientific, UK), glacial acetic acid (Fluka, Germany), and water (double distilled) were used. All stock and working solutions were freshly prepared with distilled water for HPLC analyses.

### 2.2. Hydrocortisone Pharmaceutical Preparations

Fifteen commercially available hydrocortisone pharmaceutical preparations, comprising nine tablet and six injectable formulations, were sampled from retail pharmacies within the Kumasi and Accra Metropolitan areas of Ghana. Additionally, four controlled-release hydrocortisone tablet formulations, two controlled-release capsule formulations, and one conventional release hydrocortisone tablet formulation were obtained from the Department of Pharmaceutics and Microbiology, University of Ghana School of Pharmacy, Accra, Ghana.

### 2.3. Instrumentation and Optimized Chromatographic Conditions

A chromatograph comprising a P100 Spectra Series pump with a 785A Programmable Perkin Elmer UV/VIS absorbance detector was employed in the study. Isocratic mode of elution was employed. Chromatographic separation was successfully achieved on a JT Baker® ODS C18 column (150 × 4.60 mm ID, 5 *μ*m particle size). Ultraviolet detection at a wavelength of 254 nm was able to ensure good resolution of peaks. The injection volume was 20 *μ*L and all analyses were undertaken at ambient temperature. A mobile phase consisting of methanol/water/acetic acid (60 : 30 : 10; v/v/v) at a flow rate of 1.00 ml/min was employed. All chromatographic data were acquired using PowerChrom series 280 integrator software.

### 2.4. Preparation of Standard Solution

Fifty milligrams of hydrocortisone reference standard was accurately weighed and dissolved in 25 ml of methanol as the diluent. The solution was transferred into a 50 ml dry volumetric flask and was then sonicated for 5 min. The volume was made up to the mark with methanol and mixed thoroughly (1 mg/ml stock solution). A one in ten (1 in 10) dilution was made to obtain a working solution with concentration of 0.10 mg/ml of hydrocortisone. This solution with the required working concentration was then injected to determine the retention time of the analyte of interest using the developed method.

### 2.5. Analytical Method Validation

The new HPLC method was validated in terms of linearity, limits of detection and quantification, accuracy, precision (intraday and interday), specificity, robustness, and stability, in compliance with International Conference on Harmonization (ICH) guidelines [16].

#### 2.5.1. Linearity

Serial dilutions were prepared from the stock solution of hydrocortisone reference standard (1 mg/ml) by pipetting 8.0 ml, 4.0 ml, 2.0 ml, 1.6 ml, 0.8 ml, and 0.4 ml of the stock solution into separate 20 ml volumetric flasks. The solutions were made up to volume with the diluent and mixed thoroughly. Solutions prepared were injected and analyzed using the developed method. The linearity of detector response was established by plotting a graph of concentration versus peak area of hydrocortisone standard and determining the correlation coefficient.

#### 2.5.2. Limits of Detection and Quantification

The limit of detection (LOD) and limit of quantification (LOQ) of the new HPLC method were determined from the linear regression curve obtained from the linearity of hydrocortisone. The slope and standard deviations of the responses of the linear curve were used. The formulae for the calculation of the LOD and LOQ are given below:

(1) LOD=3.3δSLOQ=10δS,

where *δ* is standard deviation of the response; *S* is slope of the calibration curve.

#### 2.5.3. Accuracy

Into three separate 10 ml volumetric flasks 1 ml of a 1 mg/ml standard solution of hydrocortisone was pipetted. 0.8 ml, 1.0 ml, and 1.2 ml of formulated hydrocortisone solution (0.1 mg/ml) were added to the three volumetric flasks, respectively, to obtain concentrations at 80%, 100%, and 120% levels. The solutions were analyzed using the developed method. Triplicate determinations were made for each solution and mean percentage recovery calculated.

#### 2.5.4. Precision

Both intraday and interday precision of the HPLC method were assessed. For intraday precision, three solutions of different concentrations were prepared in separate 20 ml volumetric flasks from the stock solution of hydrocortisone reference standard (1 mg/ml) by pipetting 8.0 ml, 2.0 ml, and 0.4 ml of the stock solution. Solutions were made up to volume with methanol. The various solutions where analyzed thrice during a particular day to obtain chromatograms from which recoveries and relative standard deviations (RSD) were calculated. For interday precision, a one in ten (1 in 10) dilution of the stock standard solution was prepared on three different occasions and analyzed. The solutions were analyzed on three consecutive days using the developed HPLC method to obtain chromatograms and the hydrocortisone content and relative standard deviation (RSD) were determined.

#### 2.5.5. Specificity

The developed HPLC method was investigated for specificity to ensure there was no or minimal interference of analyte of interest from the solvent system. The mobile phase (blank) was analyzed, followed by an injection of a sample solution of hydrocortisone. After a ten-minute wash period, the blank was analyzed again.

#### 2.5.6. Robustness

The robustness of the method was examined by introducing small changes in the composition of the mobile phase. Variation by ±10% was done with respect to the methanol component of the mobile phase while keeping the composition of the others constant. Chromatograms obtained were analyzed using the RSD of the responses obtained.

#### 2.5.7. Stability

The stability of solutions of hydrocortisone were studied over a period of 8 hours. Triplicate injections were made each hour and the chromatograms recorded.

### 2.6. Assay of Hydrocortisone Preparations

An amount of finely powdered hydrocortisone powder from each of the nine commercial conventional tablet formulations equivalent to 2.5 mg hydrocortisone was individually weighed accurately and transferred to a 25 ml volumetric flask containing 10 ml of methanol. The solution was sonicated for 5 min and made up to volume with methanol. The resulting solution was then filtered using Whatman filter paper number 41. The filtrate obtained was analyzed by making triplicate injections. The controlled-release hydrocortisone tablet and capsule formulations were analyzed in a similar way. In the analysis of the hydrocortisone injections, aliquots equivalent to 2.5 mg hydrocortisone were pipetted from the six (6) injectable preparations into 25 ml volumetric flasks and treated similarly to above. The peak areas of sample were determined and the amount of hydrocortisone was estimated from the linear regression calibration curves using the developed method. The official United States Pharmacopoeia method [11] was employed in analyzing the commercial samples. Statistical comparison between the official and developed methods was done using Student's *t*-test.

## 3. Results and Discussion 

Controlled-release hydrocortisone preparations remain formulations of choice in the therapeutic management of adrenal insufficiency disease. This disease condition is a potentially life-threatening autoimmune disorder that requires prompt diagnosis and management to avoid fatality. The use of controlled-release formulations results in more stable cortisol concentrations during the diurnal cortisol circadian pattern than conventional hydrocortisone products [17]. It is however essential that suitable and accurate analytical methods are made available to assess the quality of the products with regard to their content. The development and validation of a simple isocratic RP-HPLC method for the determination of hydrocortisone in both conventional and controlled-release pharmaceutical formulations were the focus of this study. Review of literature indicates the employment of normal phase stationary support material [18, 19] and reverse phase stationary support material [20] for the development of HPLC method and further analyses of hydrocortisone products. There is yet no reported reverse phase HPLC with UV detection method capable of analyzing hydrocortisone in both conventional and controlled-release pharmaceutical formulations. The inclusion of acetic acid as a modifier in the mobile phase system ensured efficient resolution of hydrocortisone with minimal matrix effect from both conventional and controlled-release formulations. Readily available and cost effective solvents employed in the mobile phase ensured an efficient resolution and separation of hydrocortisone on a reverse phase column.  Table 1 presents the optimized chromatographic conditions employed in the determination of hydrocortisone.

### 3.1. Analytical Method Validation

**Figure 1:**
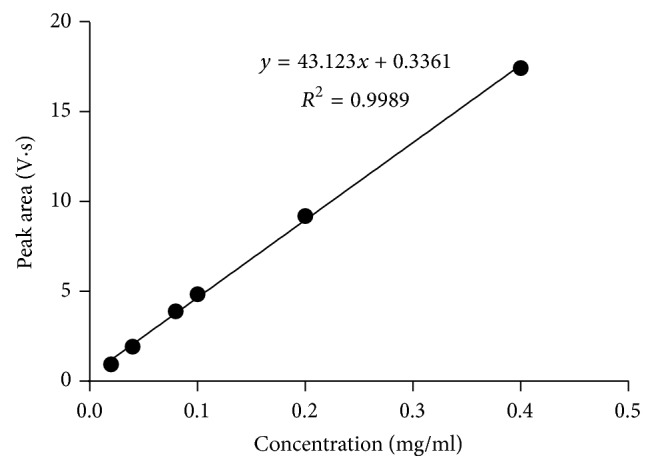
Calibration curve of hydrocortisone showing the linear detectable range (0.02–0.4 mg/ml).

**Figure 2:**
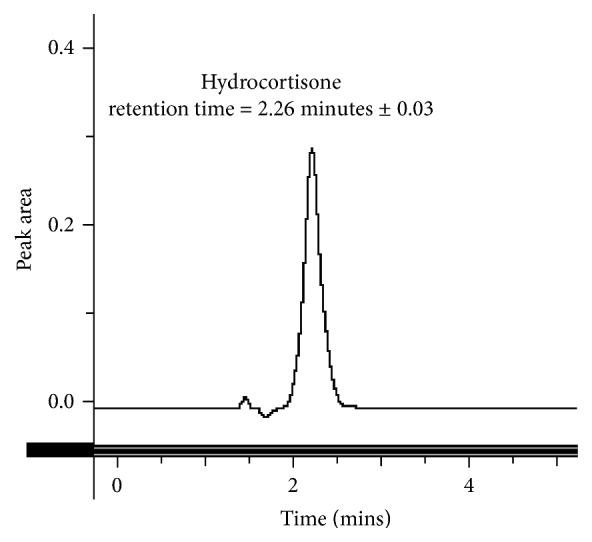
A chromatogram of hydrocortisone standard.

**Figure 3:**
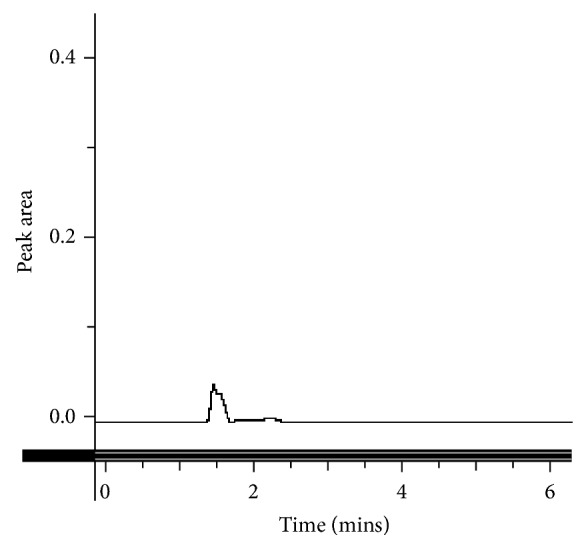
A chromatogram of an excipient in the hydrocortisone tablet.

**Figure 4:**
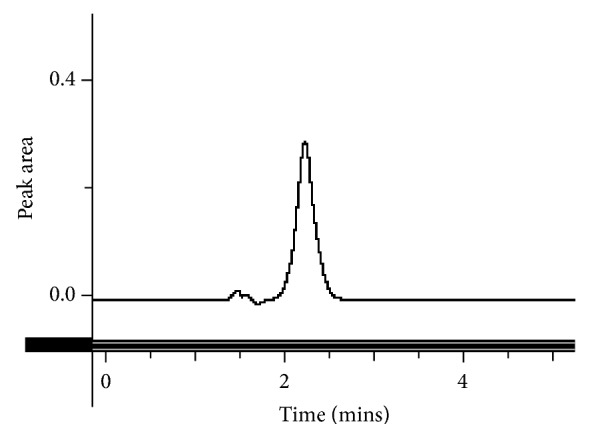
A chromatogram of the hydrocortisone tablet.

This method provides an option for the analyses of hydrocortisone in various salt forms compared to the individual methods available in some official compendia such as the United States Pharmacopoeia [11]. The analytical method being reported gave good linear detectable responses in the range of 0.02 to 0.4 mg/ml with coefficient of correlation (*r*^2^) of 0.9989 with well-resolved hydrocortisone and excipient peaks (Figures 1–4). The limit of detection and limit of quantification of 1.0662 × 10^−2 ^mg/ml and 3.23076 × 10^−2 ^mg/ml, respectively, obtained are an indication of the sensitivity of the method. The method showed good accuracy with good recoveries in the range of 98 to 101% recorded at three concentration levels of 80%, 100%, and 120% (Table 2). The method was reproducible with good intraday and interday precision of less than 1% RSD (Tables 3 and 4). The new HPLC method showed high specificity and the robustness was less than 1% RSD (Tables 5 and 6). The method also demonstrated good stability over a period of 8 h (<6% RSD) (Table 7).

### 3.2. Application of Developed Method to the Analysis of Commercial Samples

Table 8 presents the characteristics, labelled strength, and assay results of conventional hydrocortisone preparations which were analyzed using both the developed HPLC method and the official USP method. Statistical analysis (*t*-test) of data from the two methods (official/USP and developed) showed no significant difference (*p* > 0.05) at a 95% confidence interval (Table 9). The analytical method developed provides a relatively cheaper alternative technique for routine postmarket analyses of hydrocortisone formulations in resource-poor countries such as Ghana. The validated method was also successfully applied to the analysis of six controlled-release hydrocortisone tablet and capsule formulations (Table 10). The application of the method in the analyses of tablets (conventional and controlled-release) and parenteral formulations indicated the versatility of the analytical procedure. The analyses of the hydrocortisone formulations yielded percentage hydrocortisone content of 95–104%, 96–103%, and 87–103% for the four controlled-release tablet formulations, two controlled-release capsule formulations, and fifteen conventional hydrocortisone formulations, respectively. All the controlled-release products were of good quality in terms of their content (percentage of content ≥ 90%). The percentage of content of hydrocortisone powder was 100.44%. Twelve products (all 6 injections, 6 tablets) out of the 15 commercial hydrocortisone products analyzed were determined to contain the right amounts of hydrocortisone as per United States Pharmacopoeia (USP) method and the developed HPLC method [11]. This study did not cover other pharmacopoeia specifications such as disintegration and dissolution tests.

## 4. Conclusion

It can be concluded that the developed and validated simple, isocratic, sensitive RP-HPLC method is useful for the determination and quantification of hydrocortisone in conventional and controlled-release pharmaceutical formulations. The method was successfully applied to commercial samples which indicate the ability of the analytical method to distinguish between good and poor quality hydrocortisone products.

## Figures and Tables

**Table 1:** Optimized chromatographic conditions for the developed HPLC method.

Parameters	Optimized conditions
Mobile phase	Methanol/water/acetic acid (60 : 30 : 10, v/v/v)
HPLC column	JT Baker ODS C18, 5 *µ*m, 4.6 × 150 mm
Flow rate	1 ml/min
Wavelength detection	254 nm
Diluent	Methanol
Injection volume	20 *µ*L

**Table 2:** Accuracy of the HPLC method showing the mean percentage recovery of hydrocortisone at three concentration levels.

Concentration levels	Amount taken (mg/ml)	Amount added (mg/ml)	Amount recovered	Percentage recovery (%)
80%	0.10	0.08	0.07820	97.75
	0.10	0.08	0.07840	98.01
	0.10	0.08	0.07836	97.92
100%	0.10	0.10	0.10073	100.73
	0.10	0.10	0.10120	101.20
	0.10	0.10	0.10052	100.52
120%	0.10	0.12	0.11998	99.98
	0.10	0.12	0.12062	100.05
	0.10	0.12	0.11934	99.45

**Table 3:** Intraday precision of proposed HPLC method showing the mean percentage recovery and RSD of hydrocortisone at different concentrations.

Number of runs	Concentration levels (mg/ml)	Amount recovered^*∗*^ (mg/ml)	Percentage recovery (%)	% RSD
1	0.02	0.01990	99.52	0.55
2		0.01997	99.83	
3		0.01975	98.76	
1	0.10	0.10052	100.52	0.54
2		0.09945	99.45	
3		0.10000	100.03	
1	0.40	0.39952	99.88	0.19
2		0.39828	99.57	
3		0.39964	99.91	

^*∗*^Mean of three replicate determinations.

**Table 4:** Interday precision of proposed HPLC method showing the mean percentage recovery and RSD of hydrocortisone on different days.

Number of runs	Percentage recovery^*∗*^ (%)		
	Day 1	Day 2	Day 3
1	100.52	98.52	100.16
2	99.45	99.67	99.51
3	100.03	98.36	99.72
Mean	100.00	98.85	99.80
% RSD	0.54	0.71	0.33

^*∗*^Mean of three replicate determinations.

**Table 5:** Results of specificity studies of the proposed HPLC method.

Parameter	Response
*Mobile phase *	No peak (−)
Hydrocortisone	Single peak obtained
*Mobile phase*	No peak (−)

(−): not detected.

**Table 6:** Robustness of proposed HPLC method showing the mean percentage content of hydrocortisone when conditions were varied.

Sample	Original condition	Varied condition
	% content	% content
1	100.52	101.32
2	100.75	100.65
3	101.34	99.99
Mean	100.87	100.65
% RSD	0.42	0.67

**Table 7:** Stability of HPLC method showing the mean recoveries of hydrocortisone in solution over an eight-hour period.

Time (h)	Mean concentration	% RSD
0	100.72	0.56
2	100.29	0.63
4	98.40	1.73
6	95.82	3.54
8	93.11	5.79

**Table 8:** Characteristics and labelled strength of commercial hydrocortisone preparations analyzed using the developed HPLC method (DM) and the USP method.

Code	Dosage form^*∗*^	Strength	Country of manufacture	Batch number	Date of manufacture	Expiry date	Assay^*∗∗*^ (%) (DM)	Assay^*∗∗*^ (%) (USP)
HP1	PFI	100 mg	Belgium	14HB24	04/2014	03/2017	98.41	98.60
HP2	PFI	100 mg	China	131255	12/2013	12/2016	102.60	102.34
HP3	PFI	100 mg	Belgium	14HB52	12/2014	11/2017	100.64	100.42
HP4	PFI	100 mg	India	5AB01007	10/2015	09/2018	101.20	101.27
HP5	PFI	100 mg	Belgium	15HB22	09/2015	08/2018	102.80	102.83
HP6	PF1	100 mg	India	5AE04010	10/2015	09/2018	100.63	100.35
HT1	Tablet	10 mg	China	150801	08/2015	08/2018	86.80	86.45
HT2	Tablet	10 mg	France	5EH3C	05/2015	05/2018	98.90	98.94
HT3	Tablet	5 mg	France	5ET4B	04/2014	04/2017	101.55	101.72
HT4	Tablet	10 mg	India	KC004	02/2014	02/2017	101.27	101.65
HT5	Tablet	5 mg	India	KD157	04/2015	04/2018	87.69	87.36
HT6	Tablet	5 mg	India	KD150	04/2015	04/2018	89.05	89.03
HT7	Tablet	10 mg	United Kingdom	13J11/C	10/2013	10/2016	94.38	94.27
HT8	Tablet	10 mg	United Kingdom	13J14/B	10/2013	10/2016	100.28	100.11
HT9	Tablet	10 mg	United Kingdom	13J12/A	08/2013	08/2016	98.22	98.09

^*∗*^PFI = powder for injection (im/iv). ^*∗∗*^Acceptance criterion = 90–110%.

**Table 9:** Statistical comparison between the developed method and the USP method.

USP method (column B) versus developed method (column A)	
*t*-test	
*p* value	0.9742
*p* value summary	Ns
Significantly different? (*p* < 0.05)	No
One- or two-tailed *p* value?	Two-tailed
Mean ± SEM of column A	97.63 ± 1.415 *N* = 15
Mean ± SEM of column B	97.56 ± 1.440 *N* = 15
Difference between means	−0.06600 ± 2.019
95% confidence interval	−4.202 to 4.070

**Table 10:** Characteristics and labelled strength of pectin-based modified release hydrocortisone preparations analyzed using the developed HPLC method.

Code	Dosage form	Strength	Assay (%)^*∗*^
HM1	Tablet	100 mg	103.36
HM2	Capsule	100 mg	95.99
HM3	Capsule	100 mg	103.29
HM4	Tablet	100 mg	102.41
HM5	Tablet	100 mg	104.38
HM6	Tablet	100 mg	95.34

^*∗*^Acceptance criterion = 90–110 %.
